# The Importance of Timing in Immunotherapy: A Systematic Review

**DOI:** 10.7759/cureus.82994

**Published:** 2025-04-25

**Authors:** Stephanie Nagy, Atif Hussein, Marc M Kesselman

**Affiliations:** 1 Rheumatology, Kiran C. Patel College of Osteopathic Medicine, Nova Southeastern University, Davie, USA; 2 Hematology/Oncology, Memorial Cancer Institute, Pembroke Pines, USA

**Keywords:** chronotherapy, circadian rhythm, immune checkpoint inhibitors, immune system, immunotherapy, infusion time

## Abstract

Cancer remains a significant global health concern, with conventional treatments such as surgery, chemotherapy, and radiation posing considerable risks. In recent years, immunotherapy, particularly immune checkpoint inhibitors (ICIs), has emerged as a first-line treatment. ICIs enhance the immune system’s ability to target and destroy cancer cells by blocking inhibitory proteins on T-cells. The body’s circadian clock regulates essential biological functions, including immune response and metabolism, and its disruption can create an immunosuppressive environment conducive to tumor progression. Chronotherapy, which examines the influence of circadian rhythms on treatment efficacy, presents a promising approach to optimizing immunotherapy outcomes. This systematic review explores whether the timing of immunotherapy administration affects key patient outcomes, including overall survival (OS), progression-free survival (PFS), response rates, and mortality. A total of 21 studies involving 3,682 patients with a mean age of 64.7 were analyzed. Immunotherapy was administered for various cancers, the most common being melanoma, non-small cell lung cancer, and renal cell carcinoma. Only ICIs were considered, including monotherapies of nivolumab, pembrolizumab, atezolizumab, durvalumab, ipilimumab, camrelizumab, tislelizumab, sintilimab, and combination therapies of nivolumab plus ipilimumab or ICIs with additional agents such as atezolizumab with bevacizumab or pembrolizumab with axitinib. Infusion timing varied across studies, with cutoff points for early versus late administration ranging from 11:37 to 16:30, the most common being 16:30. Most studies reported improved OS and PFS in patients receiving earlier infusions, with survival benefits ranging from +2.7 to +26.6 months, with a mean of +15.1 months, and PFS extensions from -0.5 to +28.3 months, with a mean of +8.1 months. Additionally, complete and partial response rates were higher in early infusion groups. However, findings on mortality rates were inconsistent. These results suggest that the timing of immunotherapy administration may significantly impact treatment efficacy, potentially due to interactions with circadian rhythms. Further research is needed to establish standardized guidelines for optimizing infusion timing to enhance patient outcomes.

## Introduction and background

Cancer continues to be a significant public health concern worldwide, with the number of cancer diagnoses rising yearly. In 2022, more than 20 million new cancer diagnoses were made, with 9.7 million cancer-associated deaths. It has been found that 1 in 5 individuals will be diagnosed with cancer in their lifetime, with 1 in 12 succumbing to their cancer diagnosis [1]. The most frequent cancer diagnosis is lung, followed by breast, colorectal, prostate, and stomach. However, among cancer diagnoses leading to death, the most common are lung, followed by colorectal, liver, breast, and stomach [1]. Traditional therapies for cancer treatment involve surgery, chemotherapy, and radiation; however, each comes with its elevated risks, and thus, the search for alternative treatment methods began. In the last decade, a large shift has been seen in using immunotherapy as a first-line agent in cancer treatment.

Harnessing the immune system's power to be used in cancer treatment is considered a *newer* form of therapy; however, its development began in the 18th century. Due to skepticism within the medical community on the ability of medications to alter the immune system, this idea was not revisited until the 1940s [2,3]. The first approved immunotherapies for the treatment of cancer were the use of interferon-alpha as an immunomodulator for leukemia and high-dose interleukin (IL)-2 that was approved for melanoma and renal cell carcinoma [2,4]. As this field grew, the number of therapy options and utilization for immunotherapy have grown significantly. There are 6 main forms of immunotherapy treatment for cancer, including immune checkpoint inhibitors (ICI), chimeric antigen receptor T-cell therapy, monoclonal antibodies, cancer vaccines, oncolytic virus therapy, and cytokine therapy. The most common being ICI [5].

ICI targets and blocks specific proteins on the surfaces of T-cells to regulate the immune system and strengthen the attack on cancer cells. Two protein targets of ICI are cytotoxic T-lymphocyte antigen 4 (CTLA-4) and programmed cell death protein 1/programmed death-ligand 1 (PD-1/PD-L1). ICI works to inhibit the binding of CTLA-4 on T-cells and CD80/86 located on antigen-presenting cells to block the inhibition of T-cells and enhance T-cell activation and proliferation to attack cancer cells. PD-1 is expressed on T-cells, and PD-L1 is expressed on antigen-presenting cells; similarly, blocking the binding of PD-1 and PD-L1 leads to enhanced T-cell response against cancer cells [6,7]. ICI is used for a variety of cancers, including melanoma, non-small cell lung cancer (NSCLC), renal cell carcinoma, head and neck squamous cell carcinoma, urothelial carcinoma, hepatocellular carcinoma, Hodgkin lymphoma, squamous cell carcinoma, and Merkel cell carcinoma [8-11].

Immunotherapy enhances the immune system's ability to combat cancer cells; however, it is also crucial to consider the body's circadian rhythm, the biological clock that regulates immune function. Circadian clocks are present in all biological organisms. It helps them adapt to environmental changes and synchronize biological and physiological processes. When environmental light or other stimuli fluctuate according to natural laws, the central clock of the suprachiasmatic nucleus detects these changes. It then transmits signals through specific pathways, ensuring that subordinate clocks in the peripheral tissues receive the correct cues to adjust to the 24-hour cycle through hormonal and nerve signaling [12,13]. 3 main circadian loops regulate the immune system utilizing the BMAL1, CLOCK, REV-ERB, and PER and CRY genes. These genes form transcription-translation feedback loops that generate rhythmic oscillations in gene expression, which, in turn, regulate various biological activities, including the sleep-wake cycle, metabolism, hormones, immune system, and cell function. It has been found that disruptions to circadian clocks, especially those that impact the immune system and metabolism, can lead to an immunosuppressive environment, creating an environment that can support tumor growth [14,15]. To exemplify this, the International Agency for Research on Cancer has termed activities that alter circadian rhythm, such as shift work, as potentially carcinogenic to humans [16]. Animal models have shown that ablation of the suprachiasmatic nucleus or chronic jet lag in humans has led to elevated tumor growth and progression levels due to creating a beneficial microenvironment for tumor growth [14,17,18].

Chronotherapy is the study of understanding the impact that biological rhythms have on the function and effectiveness of therapies and utilizing those rhythms to optimize their actions, maximize benefits, and reduce adverse effects. Chronotherapy has begun to be explored with a variety of medications in the treatment of asthma, arthritis, cardiovascular diseases, and cancer. For example, it has been found that corticosteroids administered in the morning reduced the hypothalamic-pituitary-adrenal axis at a greater level than other times; however, corticosteroid use within multiple sclerosis patients was found to be more effective in the evening with greater clinical recovery, and had the least amount of adverse effects [19,20]. Or captopril, when administered at sleep time, has been found to improve cardiac function and reduce cardiac remodeling compared to wake-time administration [21]. Recently, harnessing the power of the circadian clock has been investigated within chemotherapy medications, which are referred to as chronomodulation chemotherapy. For example, it has been found that early administration of cyclophosphamide, doxorubicin, and rituximab leads to longer overall survival (OS) and progression-free status (PFS) in patients with diffuse large B-cell lymphoma [22]. Furthermore, the optimal chronomodulated schedules have been determined for 5-fluorouracil-leucovorin at 1 a.m. or 4 a.m., at 1 p.m. or 4 p.m. for oxaliplatin, and at 4 p.m. for carboplatin [23]. Targeting and reprogramming disrupted circadian clocks has been analyzed as a target for chemotherapy and is an effective target to utilize. This raises questions about the effectiveness of chronotherapy with immunotherapy for cancer treatment.

Recent research suggests that the body's circadian rhythm may play a crucial role in optimizing the effectiveness of immunotherapy, offering a potential avenue to enhance treatment outcomes.

Emerging studies indicate that the timing of ICI administration could impact therapeutic response. Understanding these time-dependent variations could pave the way for chronotherapy-guided immunotherapy. As a result, this systematic review aims to explore whether a certain timeframe for administering immunotherapy is more beneficial for patient outcomes of OS, PFS, mortality, and response rate.

## Review

Methodology

Search Strategy

A systematic literature review was performed using Ovid (MEDLINE), EMBASE, and Web of Science using the search terms “Immunotherapy OR Immune checkpoint Inhibitors AND Circadian rhythm OR Circadian clock OR Time-of-day.” To ensure the recency of the articles, only those published between 2014 and 2025 were assessed. The articles were analyzed in a step-wise process by first evaluating the title and abstract for relevance and then assessing the full-text manuscript. The Nova Southeastern University library database was utilized to access databases and full-text articles. 

Selection Criteria

**Figure 1:**
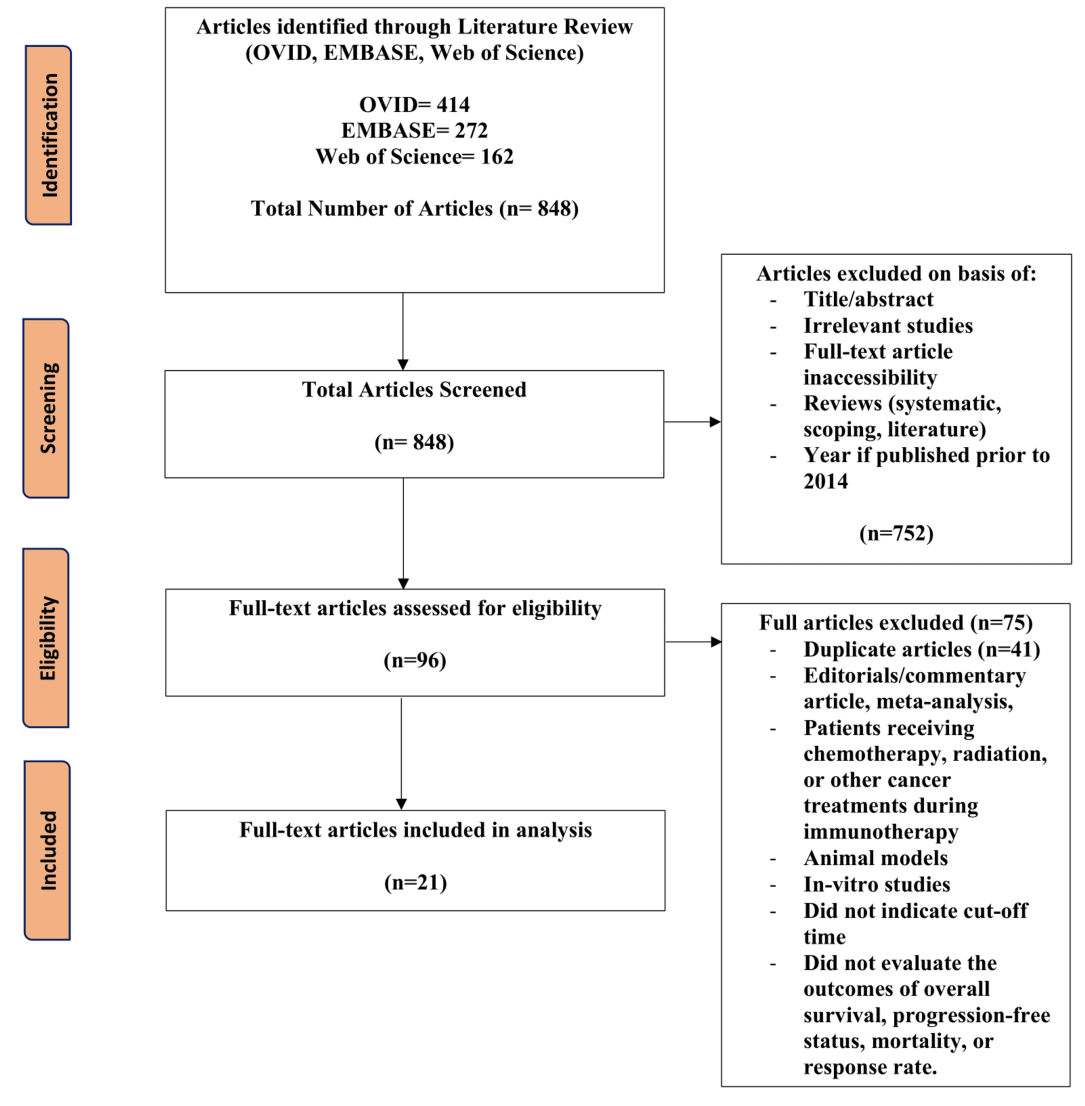
Preferred Reporting Items for Systematic Reviews and Meta-Analyses (PRISMA) indicating data selection.

For this review, we included randomized controlled trials, cross-sectional studies, observational studies, and cohort prospective/retrospective studies. The population included patients undergoing immunotherapy for cancer treatment who were not receiving other forms of cancer treatment at the time (chemotherapy or radiation). The outcome being observed is whether the timing of the therapy, earlier versus later, affected the efficacy of treatment. The observed outcomes included OS, PFS, response rate, and mortality. Studies excluded from this review were literature, systematic, or scoping reviews, animal studies, and in vitro-focused outcomes. Articles were excluded if patients were not receiving immunotherapy for cancer treatment or were simultaneously receiving other forms of cancer therapy. Studies that did not analyze the predetermined outcomes or failed to specify cutoff times distinguishing the early and late groups were also excluded. Two reviewers completed a blinded review process of the articles to decide on their inclusion or exclusion based on the determined criteria, and a third reviewer was used to break any ties. The Preferred Reporting Items for Systematic Reviews and Meta-Analyses (PRISMA) guidelines were followed and used to develop a flow diagram of the selection criteria for reproducibility (Figure 1) [24]. Risk-of-bias assessment was conducted using the Joanna Briggs Institute criteria, and all studies were found to be of adequate quality for inclusion. 

Results

Table 1 was created to display the findings within the articles, including the number, age, and sex of patients, cancer diagnosis, immunotherapy received, the timing of treatment received, and the outcomes found of OS rate, PFS rate, response rate to therapy, and mortality (Table 1).

**Table 1:** Patient characteristics and study findings.

Author	Number of patients and Sex (M = male, F = female)	Mean age (years)	Cancer type	Treatment received	Timing of treatment	Overall survival (OS) findings	Progression-free status (PFS) findings	Response rate findings	Mortality rate
Iwahashi et al. (2024) [25]	257 (did not specify sex)	Not specified	Non-small cell lung cancer	Nivolumab	The early group received treatment before noon, and the late group received treatment after 12:00	16.9 months in the early group versus 14.2 months in the late group (*P* < 0.409)	4.2 months in the early group versus 4.7 months in the late group (*P* < 0.554)	Not analyzed	Not analyzed
Gonzalez et al. (2024) [26]	227 F	Not specified	Gynecological cancers impacting the endometrium (*n* = 104), ovarian (*n* = 56), cervical (*n* = 43), vulva (*n* = 2), vagina (*n* = 6)	Not specified	The early group received treatment before 16:00, and the late group received treatment after 16:00	19.3 months in the early group versus 10.3 months in the late group (*P* < 0.0005)	6.8 months in the early group versus 3.1 months in the late group (*P* < 0.002)	Patients who received the majority of treatments before 16:00 had a statistically significant improvement in partial and complete response (*P* < 0.0030)	Not analyzed
Huang et al. (2019) [27]	183 (M = 97, F = 23)	62	Esophageal cancer	Camrelizumab (*n* = 44), pembrolizumab (*n* = 29), tislelizumab (*n* = 18), sintilimab (*n* = 27)	The early group received patients who received the majority of treatment at noon, and the late group received patients who received the majority of treatment in the afternoon.	Patients who had the majority of treatment after noon had significantly improved OS (*P* < 0.013). Exact months were not provided.	Not analyzed	Receiving all infusions after noon was significantly associated with a higher pathological complete response rate (*P* = 0.040)	Not analyzed
Pascale et al. (2024) [28]	131 (M = 119, F = 12)	70	Hepatocellular carcinoma	Atezolizumab with bevacizumab	The early group is patients who received one or both cycles before 13:00, and the late group is patients who received both cycles after 13:00.	18.7 months in the early group versus 11.5 months in the late group (*P* < 0.015)	Not analyzed	Not analyzed	Not analyzed
Nomura et al. (2023) [29]	62 (M = 50, F = 12)	Not specified	Esophageal squamous cell carcinoma	Nivolumab	The early group is patients who received treatment before 13:00, and the late group is patients who received treatment after 13:00.	60% in the early group and 56.8% in the late group after one year (*P* < 0.036)	23.0% in the early group and 11.1% in the late group after one year (*P* < 0.303)	Overall response rate was 44.4% in the early group and 20.0% in the late group (*P* = 0.038). Disease control rate was 51.9% in the early group and 34.3% in the late group (*P* = 0.165)	Not analyzed
Dizman et al. (2023) [30]	135 (M = 94, F = 41)	64	Metastatic renal cell carcinoma	Nivolumab and ipilimumab (*n* = 69) or nivolumab monotherapy (*n* = 66)	The early group is patients who received treatment before 16:30, and the late group is patients who received treatment after 16:30.	Significantly higher in the early treatment group versus the late group (*P* < 0.016)	Not analyzed	Overall response rate was 36.0% in the early group as compared to 29.5% in the late group (*P* = 0.157). Complete response rate was 7.9% in the early group and 2.3% in the late group. Nonsignificant increase in overall response rate and complete response rate in patients treated with monotherapy nivolumab versus nivolumab and ipilimumab.	Not analyzed
Catozzi et al. (2024) [31]	361 (M = 222, F = 139)	62.5	Non-small cell lung cancer (*n* = 289), colorectal carcinoma (*n* = 3), melanoma (*n* = 18), ear nose throat cancer (*n* = 22), breast cancer (*n* = 1), urinary cancers (*n* = 27), pancreatic cancer (*n* = 1)	Atezolizumab (*n* = 20), durvalumab (*n* = 22), nivolumab (*n* = 132), pembrolizumab (*n* = 186), ipilumumab (*n* = 1)	The early group is patients who received treatment before 11:37, and the late group is patients who received treatment after 11:37.	30.3 months in the early group versus 15.9 months in the late group (*P* < 0.0024)	Not analyzed	Partial and complete response rates were higher in the early group at 58% versus 41% in the late group (*P* < 0.027)	Mortality rate increased in the late infusion group (*P* = 0.003)
Goncalves et al. (2023) [32]	78 (M = 45, F = 33)	64	Metastatic melanoma	Nivolumab or pembrolizumab, or nivolumab plus ipilimumab	The early group is patients who received the majority of treatment before 14:00, and the late group is patients who received treatment after 14:00.	38.1 months in the early group versus 14.2 months in the late group (*P* < 0.001)	14.9 months in the early group versus 6.6 months in the late group (*P* < 0.320)	Not analyzed	Not analyzed
Rousseau et al. (2023) [33]	180 (M = 112, F = 68)	65	Non-small cell lung cancer	Nivolumab or pembrolizumab, or atezolizumab	The early group is patients who received the majority of treatment before 16:30, and the late group is patients who received the majority of treatment after 16:30	26.2 months in the early group versus 14 months in the late group (*P* < 0.090)	9.4 months in the early group versus 4.9 months in the late group (*P* < 0.0020)	Not analyzed	Mortality rate was not elevated in the late group (*P* < 0.055)
Cortellini et al. (2022) [34]	262 (M = 131, F = 131)	69	Non-small-cell lung cancer	Pembrolizumab	The early group is patients who received the majority of treatment before 16:30, and the late group is patients who received the majority of treatment after 16:30	47.1 months in the early group versus 27.8 months in the late group (*P* = 0.11)	19.7 months in the early group versus 6.6 months in the late group (*P* = 0.056)	Not analyzed	Not analyzed
Patel et al. (2024) [35]	201 (M = 146, F = 55)	63	Renal cell carcinoma	Nivolumab or nivolumab plus ipilimumab or pembrolizumab	The early group is patients who received the majority of treatment before noon, and the late group is patients who received the majority of treatment after 12:00	Early infusion time had a significantly longer OS (*P* = 0.033) – exact months were not provided	Early infusion time had significantly longer PFS (*P* = 0.020) – exact months were not provided	Response rate was elevated in the early group at 34% compared to the late group at 21% (*P* = 0.044).	Not analyzed
Goncalves et al. (2024) [36]	168 (M = 100, F = 68)	69	Melanoma	Ipilimumab with nivolumab, or nivolumab, or pembrolizumab	The early group is patients who received the majority of treatment before 14:00, and the late group is patients who received the majority of treatment after 14:00	37.6 months in the early group versus 14.4 months in the late group (*P* < 0.014)	PFS did not notice a statistically significant increase between groups - exact months were not provided.	Not analyzed	Not analyzed
Molina-Cerrillo et al. (2022) [37]	61 (did not specify sex)	Not mentioned	Renal cell carcinoma	Nivolumab with ipilimumab (*n* = 46), pembrolizumab with axitinib (*n* = 15)	The early group is patients who received the majority of treatment before 16:30, and the late group is patients who received the majority of treatment after 16:30	Not analyzed	12.3 months in the early group versus 5.6 months in the late group (*P* = 0.048)	Greater overall survival in the early group, but not statistically significant (*P* = 0.16)	Not analyzed
Fletcher et al. (2025) [38]	516 (M = 329, F = 187)	61.1	Melanoma	Nivolumab or pembrolizumab, or a combination of ipilimumab/PD-1 inhibitor	The early group is patients who received the majority of treatment before 16:00, and the late group is patients who received the majority of treatment after 16:00	81.2 months in the early group versus 54.6 months in the late group (*P* = 0.19)	38.9 months in the early group versus 10.6 months in the late group (*P* = 0.001)	Overall response rate in the early group was 46.9% compared to 31.9% in the late group (*P* = 0.001).	Not analyzed
Janopaul-Naylor et al. (2024) [39]	62 (did not specify sex)	63.66	Head and neck squamous cell carcinoma	Nivolumab	Morning group is patients who received the majority of treatment before 11:00 (*n* = 7), midday group is those who received the majority of treatment between 11:00 and 16:30 (*n* = 34), and afternoon group is patients who received the majority of treatment after 16:30 (*n* = 21)	Morning group was 14 months, midday group was 14.5 months, and afternoon group was 14.4 months (*P* > 0.9)	Morning group was 5.8 months, midday group was 2.9 months days and afternoon group was 1.8 months (*P* = 0.8)	Overall response rate for the morning group was 57%, midday group was 33%, and afternoon group was 25% (*P* = 0.2).	Not analyzed
Ramirez et al. (2024) [40]	127 (M = 90, F=37)	62	Renal cell carcinoma	Nivolumab plus Ipilimumab	The early group is patients who received the majority of treatment before 16:30, and the late group is patients who received the majority of treatment after 16:30	64.8 months in the early group versus 46.3 months in the late group (*P* = 0.03)	Not analyzed	Overall response rate was greater in the early group at 32.8% compared to 22.4% (*P* = 0.04).	Not analyzed
Karaboue et al. (2024) [41]	95 (M = 79, F = 16)	66.9	Non-small cell lung cancer	Nivolumab	The early group is patients who received the majority of treatment between 09:27-12:54, and the late group is patients who received the majority of treatment between 12:55 and 17:14	34.2 months in the early group versus 9.6 months in the late group (*P* < 0.001)	Not analyzed	Overall response rate was greater in the early group at 37.5% versus 14.4% in the late group (*P* < 0.029). Disease control rate was higher in the early group at 77.1% compared to 50.0% in the late group (*P* = 0.006)	Not analyzed.
Ruiz-Torres et al. (2024) [42]	113 (M = 78, F = 33)	65	Head and neck cancer	Pembrolizumab or nivolumab or ipilimumab or durvalumab	The early group is patients who received the majority of treatment before 15:00, and the late group is patients who received the majority of treatment after 15:00	Longer OS in the early group versus the late group (*P* < 0.26) - months were not provided	Longer PFS in the early group versus the late group (*P* < 0.04) - months were not provided	Not analyzed	Not analyzed
Tanaka et al. (2024) [43]	58 (M = 48, F = 10)	67	Gastric cancer	Nivolumab	The early group is patients who received the majority of treatment between 9:40- 11:40, and the late group is patients who received the majority of treatment after 12:20-15:30	8.2 months in the early group versus 5.4 months in the late group (*P* < 0.001)	2.6 months in the early group versus 1.6 months in the late group (*P* < 0.004)	Overall response rate was elevated in the early group at 17.2% months compared to 3.4% months in the late group; Disease control rate was elevated in the early group at 48.3% months compared to 31.0% months in the late group.	Not analyzed
Janse Van Rensberg et al. (2022) [44]	106 (did not specify sex)	Not specified	Head and neck squamous cell carcinoma (*n* = 19); breast cancer (*n* = 22), ovarian (*n* = 21), melanoma (*n* = 12), other (*n* = 32)	Pembrolizumab	The early group is patients who received the majority of treatment before 16:30, and the late group is patients who received the majority of treatment after 16:30	No differences were noted - months were not specified	No differences were noted - months were not specified	Not analyzed	Not analyzed
Qian et al. (2021) [45]	299 (M = 197, F = 102)	61	Melanoma	Ipilimumab, pembrolizumab, nivolumab, or a combination	The early group is patients who received the majority of treatment before 16:30, and the late group is patients who received the majority of treatment after 16:30	57.6 months in the early group versus 46.8 months in the late group (*P* = 0.038)	Increase in the early group at 56% versus 40% in the late group (*P* < 0.041)	Increased complete response rate in the early group at 34% versus 22% in the late group (*P* < 0.069)	Not analyzed

**Figure 2:**
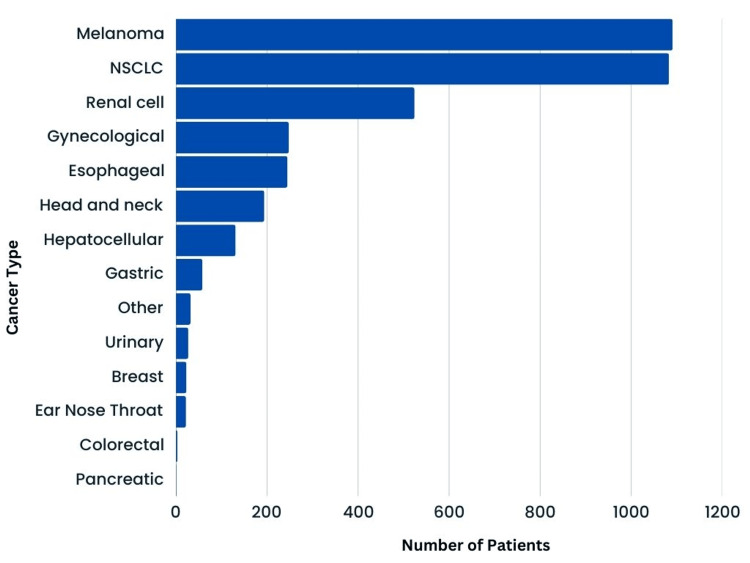
Cancer types treated with immunotherapy. NSCLC, non-small cell lung cancer

Within the 21 studies included, a total of 3,682 patients were analyzed; however, the sex of 549 patients was not disclosed. Of the remaining 3,133 patients, for whom the sex was disclosed, 1,194 were female and 1,939 were male. Of the 21 studies, 16 reported the average patient age, which ranged from 61.1 to 70 years, with a mean age of 64.7 years. Immunotherapy was prescribed for a variety of cancers with the most common being melanoma (*n *= 1,091, 29.6%), followed by NSCLC (*n *= 1,083, 29.4%), renal cell carcinoma (*n *= 524, 14.2%), gynecological cancers (*n *= 248, 6.7%), esophageal carcinoma (*n *= 245, 6.7%), head and neck cancer (*n *= 194, 5.3%), hepatocellular carcinoma (*n* = 131, 3.6%), gastric cancer (*n *= 58, 1.6%), other (*n *= 32, 0.9%), urinary cancers (*n *= 27, 0.7%), breast cancer (*n *= 23, 0.6%), ear nose throat cancer (*n *= 22, 0.6%), colorectal carcinoma (*n *= 3, 0.06%), and pancreatic cancer (*n *= 1, 0.04%) (Figure 2).

Type of Immunotherapy Used

Immunotherapy treatment options in the included studies were limited to immune checkpoint inhibitors (ICIs), either as monotherapy with agents such as nivolumab, pembrolizumab, atezolizumab, durvalumab, ipilimumab, camrelizumab, tislelizumab, or sintilimab, or as combination therapies, including nivolumab plus ipilimumab and regimens combining ICIs with other agents, such as atezolizumab with bevacizumab (a VEGF inhibitor) or pembrolizumab with axitinib (a tyrosine kinase inhibitor). Studies did not consistently provide the number of patients receiving each form of therapy, making it difficult to analyze which therapy would have been the most effective; however, qualitatively, nivolumab was used to a greater extent between the studies.

Timing of Immunotherapy 

The timing of treatment varied considerably across studies, particularly in terms of the cutoff points used to define early and late treatment groups. Reported cutoff times ranged from 11:37 to 16:30. The most common cutoff time used was 16:30 (*n *= 7) to separate between the early and late groups followed by noon (*n *= 3), 13:00 (*n* = 2), 16:00 (*n* = 2), 14:00 (*n* = 2), 11:37 (*n *= 1), 11:40 (*n *= 1), 12:55 (*n *= 1), 15:00 (*n *= 1), and one study separate groups into morning 11:00, midday 11:00-16:30 and afternoon after 16:30 (*n *= 1). As a result of this, we further classified the articles into three groups based on the most common infusion time frames to further compare the findings: those with a cutoff time before 13:00 (*n *= 9, Table 2), those with a cutoff time between 16:00 and 16:30 (*n *= 9, Table 3), and finally, those with a cutoff time between 13:00 and 16:00 (*n *= 3, Table 4).

**Table 2:** Articles with infusion time cutoffs before 13:00.

Author	Cutoff time	General findings of OS	General findings of PFS	General findings of the response rate	General findings of mortality
Iwashashi et al. (2024) [25]	12:00	Non-significant improvement	Non-significant improvement	Not analyzed	Not analyzed
Huang et al. (2019 [27]	12:00	Significant reduction	Not analyzed	Significant reduction	Not analyzed
Pascale et al. (2024) [28]	13:00	Significant improvement	Not analyzed	Not analyzed	Not analyzed
Nomura et al. (2023) [29]	13:00	Significant improvement	Significant improvement	Significant improvement	Not analyzed
Catozzi et al. (2024) [31]	11:37	Significant improvement	Not analyzed	Significant improvement	Significant improvement
Patel et al. (2024) [35]	12:00	Significant improvement	Significant improvement	Significant improvement	Not analyzed
Janopaul-Naylor et al. (2024) [39]	11:00	Non-significant improvement	Non-significant improvement	Non-significant improvement	Not analyzed
Karaboue et al. (2024) [41]	12:55	Significant improvement	Not analyzed	Significant improvement	Not analyzed
Tanaka et al. (2024) [46]	12:20	Significant improvement	Significant improvement	Significant improvement	Not analyzed

**Table 3:** Articles with infusion time cutoffs between 16:00 and 16:30.

Author	Cutoff time	General findings of OS	General findings of PFS	General findings of response rates	General findings of mortality
Gonzalez et al. (2024) [26]	16:00	Significant improvement	Significant improvement	Significant improvement	Not analyzed
Dizman et al. (2023) [30]	16:30	Significant improvement	Not analyzed	Non-significant improvement	Not analyzed
Rousseau et al. (2023) [33]	16:30	Significant improvement	Significant improvement	Not analyzed	Non-significant elevation
Cortellini et al. (2022) [34]	16:30	Non-significant improvement	Non-significant improvement	Not analyzed	Not analyzed
Molina-Cerrillo et al. (2022) [37]	16:30	Not analyzed	Significant improvement	Non-significant improvement	Not analyzed
Fletcher et al. [38]	16:00	Non-significant improvement	Significant improvement	Significant improvement	Not analyzed
Ramirez et al. (2024) [40]	16:30	Significant improvement	Not analyzed	Significant improvement	Not analyzed
Janse Van Rensberg et al. (2022) [44]	16:30	Non-significant improvement	Non-significant improvement	Not analyzed	Not analyzed
Qian et al. (2021) [45]	16:39	Significant improvement	Significant improvement	Non-significant improvement	Not analyzed

**Table 4:** Articles with infusion time cutoffs between 13:00 and 16:00.

Author	Cutoff time	General findings of OS	General findings of PFS	General findings of the response rate	General findings of mortality
Goncalves et al. (2022) [36]	14:00	Significant improvement	Non-significant improvement	Not analyzed	Not analyzed
Goncalves et al. (2024) [32]	14:00	Significant improvement	Non-significant improvement	Not analyzed	Not analyzed
Ruiz-Torres et al. (2024) [42]	15:00	Non-significant improvement	Significant improvement	Not analyzed	Not analyzed

Outcome Analysis

OS and PFS were common outcomes within the studies analyzed. Almost all studies that examined these outcomes found the earlier groups to have a greater OS and PFS than the later groups. OS rate is the number of months the patient survived following treatment, and PFS refers to the number of months the patient’s condition remained stable.

Within the studies analyzed, 20 were assessed for OS. Out of those studies, all found that early infusion times increase OS, except for Huang et al. and Janse Van Rensberg et al. [27,44]. Huang et al. found that the latter group had a greater OS; however, this was opposite to all other studies, and Janse Van Rensberg et al. found no significant change [27,44]. Analyzing the studies based on their groupings (Tables 2, 3, 4), those with cutoff times before 13:00 all had an improvement in their OS for patients who received their therapy early, with only Huang et al. indicating opposite findings (Table 2). Furthermore, the majority noted these findings to be a significant improvement. In the 16:00 to 16:30 group, 8 of the 9 studies analyzed OS, all found an increase in OS in patients who received their therapy earlier (5 out of the 8 noting significantly improved OS) (Table 3). Finally, those receiving therapy between 13:00 and 16:00 all found improvements in OS (2 out of 3 noting significant improvements in OS) (Table 4). In total, of the 13 out of 20 studies that reported data for the number of months of OS, on average, early infusion time had a greater number of months of survival that ranged from +2.7 to +26.6 months, with a mean of 15.1 months. Meanwhile, Nomura et al. noted a 3.2% increase in OS in the early group, and Dizman et al., Ruiz-Torres et al., and Patel et al. noted statistically significant increases without providing exact months.

PFS was analyzed within 15 studies. In total, all studies concluded a greater PFS in early infusion groups compared to the late groups, except for Iwahashi et al., who concluded a non-statistically significant decrease of only 0.5 months, and Janse Van Rensberg et al., who did not note any difference between the groups. However, they did not provide further analysis. Analyzing the studies based on their groupings (Tables 2, 3, 4), 5 out of the 9 with cutoff times before 13:00 analyzed PFS (Table 2). Each indicated an improvement of PFS in the early group (3 out of the 5 indicated statistically significant findings). In the 16:00 to 16:30 group, 7 of the 9 studies analyzed PFS, all found an increase in PFS in patients who received their therapy earlier (5 out of the 8 noting significant improvements) (Table 3). Finally, those receiving therapy between 13:00 and 16:00 all found improvements in PFS (1 out of the 3 noting significant improvements) (Table 4). When analyzing all of the articles, only 8 of the 13 studies reported data for the number of months of PFS between the two groups, ranging from -0.5 to +28.3 months, with a mean of 8.1 months. Meanwhile, Qian et al. and Nomura et al. measured PFS as a percentage and noted a 16% and 11.9% increase in PFS, respectively. Of the studies that did not report values, Goncalves et al. noted a non-statistically significant increase, while Patel et al. noted a statistically significant increase.

In patients in studies with cutoff times before 13:00, 7 out of 9 analyzed response rates. All noted improvements in response rates, except for Huang et al., the only study in the entire review that noted a reduction in response rate. Patients who received infusions with a cutoff time of after 16:00, 6 out of the 9 studies analyzed, showed complete and overall response rates; all noted an increase in the response rates (Table 3). Response rate was not monitored for any patients who received therapies between 13:00 and 16:00. In conclusion, Gonzalez et al. and Catozzi et al. found that those in the early group had a statistically significant improvement in both partial and complete response, while Dizman et al. and Qian et al. only analyzed the complete response rate and similarly found an elevated complete response rate in the early group versus the late group. Noumura et al., Patel et al., Fletcher et al., Janopaul-Naylor et al., Dizman et al., Ramirez et al., Karaboue et al., and Tanaka et al. all analyzed the overall response rate, and each study concluded that the early group had an improvement in overall response rate compared to the late group.

Nourmura et al., Karaboue et al., and Tanaka et al. found a greater disease control rate in the early group than in those receiving later infusions.

Mortality rates were only analyzed within three studies; however, they found conflicting evidence. Rousseau et al. found that mortality rates did not increase in the late group (*P *< 0.055), but this was not statistically significant. Conversely, Catozzi et al. and Molina-Cerrillo et al. did find a statistically significant increase in mortality rates within the late infusion group (*P *= 0.003) and a greater survival rate within the early group recipients (*P *= 0.16), respectively. It is important to note that the cutoff time between the studies did vary, with Catozzi et al. having the earliest cutoff time at 11:37 versus Rousseau et al. and Molina-Cerrillo et al., both using 16:30 as the cutoff time but finding opposite results in mortality risk.

Discussion

Immunotherapy has become a staple treatment for a variety of cancers. Compared to traditional methods, it allows for a more specific approach to activating the body's immune system to target an attack on the cancer cells, which has been found to result in fewer side effects, longer-lasting remission time, and more tailored treatment for patients [47]. However, recent studies have been investigating the use of the circadian clock in improving the efficacy of certain medications, leading to an expansion of literature in the field of chronotherapy. There has been a shift to understanding how immunotherapy and its timing may impact/coincide with the body's natural control over the immune system, as a result, this review aimed to better understand the relationship between the initiation of immunotherapy and patient outcomes. 

It is crucial to better understand the various aspects of the immune system and its regulation, to gain a greater understanding of how timing and immunotherapy may affect these systems, as a dysfunction of them can lead to an elevated risk of tumor development, exacerbated inflammatory response, and autoimmunity [48]. It has been found that 3 circadian loops regulate the immune system utilizing the BMAL1, REV-ERB, and PER and CRY genes. Within the first loop, the BMAL1 gene is used to regulate lymphatic endothelial cells and dendritic cells in the skin by activating chemokine receptors of C-C chemokine receptor type 7 (CCR7) and C-X-C chemokine receptor 4 (CXCR4) on the surface of these cells. This is essential to allow for the migration of white blood cells and dendritic cells into the lymphatic system from the peripheral tissues [49,50]. BMAL-1 can also bind to promoter regions of chemokines, regulating their transcriptional activity, which helps guide immune cells to enter the lymphatic system in a time-dependent manner. If BMAL-1 is knocked out, it has been found that immune cells are unable to enter the lymphatic system, inhibiting this migration [50,51]. BMAL1 has also been found to induce pro-inflammatory and antioxidant factors in response to signaling from the circadian clock [50]. The second loop involved the REV-ERB genes, a part of the nuclear receptor superfamily. REV-ERB are transcriptional regulators and, thus, are expressed in dendritic cells, macrophages, and T-cells. REV-ERB reduces pro-inflammatory cytokines of IL-1, nucleotide-binding domain, leucine-rich-containing family, pyrin domain-containing-3 (NLRP3) inflammasome, intra-cellular caspase-1, and chemokine (C-C motif) ligand 2 (CCL2). This can help in the treatment of acute and chronic inflammatory diseases. BMAL1 gene and REV-ERB gene loops have been found to intertwine, as BMAL1 can induce an inflammatory cascade during infections, and REV-ERB transcription factors have been found to regulate the inflammatory response downstream from BMAL1 [50,52]. Together, these loops can regulate the circadian rhythmicity of immune cell physiological processes. The third loop is regulated by the PER and CRY genes, which have been found to have an important role in controlling complex feedback oscillation. Specifically, they are found to inhibit the BMAL1 gene interaction with the CLOCK gene to achieve rhythmic feedback loops [50,53]. Other than controlling the circadian loops, they have more recently been found to be crucial links between circadian disruption and disease. For example, in lung adenocarcinoma cases, PER is downregulated, potentially indicating its use as a tumor suppressor agent [50,53]. Also, it was concluded that PER-3 polymorphism has been found to result in shorter PFS [54]. 

The circadian system plays a crucial role in maintaining homeostasis; however, disruptions in this can lead to infections, autoimmune conditions, and cancers. Since the disruptions of circadian rhythm have been identified as potential carcinogens, further investigation has been conducted to better understand this association. Cancer cells can benefit from this dysregulation; however, they also have been found to utilize their mechanism to hijack the body's circadian rhythms. Inactivation of BMAL1 has been shown to result in the rapid proliferation of hematologic cancers, colon cancer, pancreatic cancer, tongue squamous cell carcinoma, breast cancer, lung adenocarcinoma, hepatocellular carcinoma, nasopharyngeal carcinoma, and glioblastoma. Oppositely, overexpression of BMAL1 has been found to regulate and suppress cancer cells through activating cell cycle arrest and apoptosis. Regarding the PER-CRY genes, the PER anticancer mechanism stems from inhibiting the phosphoinositide 3 kinase (PI3K)/protein kinase B (AKT)/mammalian target of rapamycin (mTOR) pathway, which inhibits glycolysis in cancer cells, limiting their growth and resulting in arrest and apoptosis. For example, specifically within breast cancer patients, it was concluded that tumor growth occurred in a cyclical nature with two peaks of optimum growth that coincided with downregulated levels of PER [55]. CRYs have been found to degrade pro-oncogenes of cellular MYC (cMYC), early region 2 binding factor (E2F), and tousled-like kinase 2 (TLK2) by recruiting the ubiquitin-ligase complex. As such, the downregulation of PER and CRY can result in a suitable environment for cancer growth [56]. Having a better understanding of how dysregulation of these circadian genes can lead to cancers highlights the importance of utilizing these circadian loops with proper immunotherapy timing to inhibit the growth and proliferation of cancer cells.

It is also crucial to analyze the major genes influencing the biological clock and the impact that the time of day can have on the immune system, as this can further assist in identifying the best timing for immunotherapy. Circadian clocks regulate most of the physiological processes in the body over 24 hours; however, it has been shown that following these rhythms, the activation of the immune system is modulated according to the time of day and is more active within the morning hours [49]. Dendritic cells and CD8 T-cells are crucial for anti-tumor immunity and follow a circadian rhythmic pattern that is more active in the morning. In the morning, there is a greater elevation of CD80, a co-stimulator, that influences greater drainage of dendritic cells into the lymph nodes, leading to activation of the CD8 T-cells [49,53,57]. This was further confirmed if an anti-CD80 antibody was introduced or if the BMA1 gene, which regulates CD80's expression, was dysfunctional; it led to dysfunction and loss of time-of-day stimulation of CD8 T-cells [53,57]. It was also found that CD8 T-cells followed the rhythm of epinephrine release, which elevates in the morning and decreases in the evening [58]. Furthermore, when analyzing tumor microenvironments, it was found that there is a significantly elevated number of CD4 and CD8 T-cells within tumors during the morning; it is said that the immune system has a higher anti-tumor ratio within the morning hours [53,57]. Furthermore, the production of B lymphocytes was seen to be elevated during the first activity, which in humans is commonly in the morning hours; this is when the highest number of lymphocytes are observed following a rhythmic pattern of homing lymphocytes from the blood into the lymph nodes and vessels [59]. Furthermore, CCR7 has a diurnal oscillation pattern that peaks in the morning, influencing an elevation of both T- and B-cells [59].

Understanding the earlier peak trends of key components of the immune system provides potential evidence as to why almost all articles noticed elevated OS, PFS, or response rate within patients who received their immunotherapy within the earlier groups. The elevation of dendritic cell migration, activation of CD4 and especially CD8 T-cells in the earlier hours, in addition to the added benefit of ICI inhibiting the inactivation of T-cells, leading to greater proliferation and activation, provides a strong immunological response against cancer cells. The natural elevation of T-cells follows the body's circadian rhythm, plus the added benefit of ICI further strengthening T-cell numbers, may be the combination that is required to lead to stronger anti-tumor activity.

It is important to note that everyone has a slight modification within their circadian rhythm timing. Within the findings of the study, overall, the earlier groups obtained greater results, but the cutoff times greatly varied. The most common cutoff time was 16:30; however, it ranged from 11:37 to 16:30. Traditionally, morning is thought of to be from 06:00 to 12:00, afternoon from 12:00 to 18:00, evening from 18:00 to 20:00, and night from 21:00 to 05:00. The cutoff times within the studies was split between morning (*n *= 2) and afternoon (*n *= 19). It may be hypothesized that an *early* infusion time can be anytime within the morning or afternoon from 06:00 to 18:00, as beneficial results were seen during all timeframes within these hours (Tables 2, 3, 4). It is recommended for future studies to truly analyze cutoff times by conducting a large-scale randomized controlled trial that places patients in morning, afternoon, and evening infusion times to better understand if a variation exists in immunotherapy efficacy when given at different times.

Future research should establish a standardized cutoff time to enhance the study's reliability and applicability to distinguish early versus late immunotherapy administration. This would allow for a clearer understanding of the optimal timing for maximizing patient benefits. Additionally, incorporating detailed data on ICI dosage, frequency, and the number of patients receiving each type of ICI would provide valuable insights into the comparative efficacy of different ICIs and the most effective dosing regimens. Expanding the study population to include a more diverse representation of cancer types, rather than predominantly melanoma and NSCLC cases, would improve the generalizability of findings. Furthermore, ensuring comprehensive reporting of cancer stage and duration would allow for a more nuanced analysis of treatment outcomes across different disease progressions. Further research is essential to establish standardized guidelines for optimizing immunotherapy infusion timing and improving patient outcomes. While existing studies suggest that earlier administration may enhance OS, PFS, and response rates, variations in study designs, cancer types, and cutoff times highlight the need for more rigorous, large-scale clinical trials. As within this study, there was a wide range of infusion time cutoffs from 11:37 to 16:30, even though similar results were found when analyzed in groupings, additional research should be conducted to hone in on one specific time frame that is the most effective. Future research should focus on identifying the optimal infusion window based on circadian biology, tumor characteristics, and individual patient factors. Standardized protocols could help personalize treatment schedules, maximize therapeutic efficacy, and minimize adverse effects, ultimately advancing precision medicine in oncology. Finally, conducting a meta-analysis is crucial to better understand the statistical power and overall robustness of the findings.

## Conclusions

The influence of the circadian rhythm on the immune system is only beginning to be understood. The timing of immunotherapy infusion may play a significant role in optimizing treatment outcomes for cancer patients, potentially influencing efficacy due to the body’s circadian rhythm. While current evidence suggests that earlier infusion times, particularly in the morning and afternoon, may enhance treatment benefits, leading to longer OS, PFS, and greater response to therapy, inconsistencies in cutoff times across studies highlight the need for further investigation. Given the emerging understanding of circadian influences on the immune system, future large-scale randomized controlled trials should be conducted to systematically assess the impact of morning, afternoon, and evening infusions on immunotherapy efficacy. Establishing an optimal infusion time could lead to improved patient outcomes and more personalized treatment strategies in oncology.
